# Association between Sedentary Time and Falls among Middle-Aged Women in Japan

**DOI:** 10.3390/healthcare10122354

**Published:** 2022-11-23

**Authors:** Etsuko Ozaki, Daisuke Matsui, Nagato Kuriyama, Satomi Tomida, Yukiko Nukaya, Teruhide Koyama

**Affiliations:** 1 Department of Epidemiology for Community Health and Medicine, Graduate School of Medical Science, Kyoto Prefectural University of Medicine, Kyoto 602-8566, Japan; 2 Division of Social Health Medicine, Shizuoka Graduate University of Public Health, Shizuoka 420-0881, Japan; 3 Division of Endocrine and Breast Surgery, Department of Surgery, Graduate School of Medical Science, Kyoto Prefectural University of Medicine, Kyoto 602-8566, Japan; 4 Department of Dental Medicine, Graduate School of Medical Science, Kyoto Prefectural University of Medicine, Kyoto 602-8566, Japan

**Keywords:** fall risk, sedentary time, healthy life, daily physical activity

## Abstract

There are many reports on the risk of falls in older adults but none regarding the risk among middle-aged people. We aimed to determine fall risk factors among middle-aged women. The participants comprised 1421 women aged 40 to 64 years; anthropometric and other measurements were obtained, and lifestyle factors were examined using a self-administered questionnaire. The participants were categorized into two groups (No-fall and Fall/Almost-fall) based on their questionnaire responses. The No-fall and Fall/Almost-fall groups comprised 1114 and 307 participants, respectively. Body mass index, abdominal circumference measurements, and prevalence of dyslipidemia were significantly higher in the Fall/Almost-fall group. Additionally, those in the Fall/Almost-fall group had a shorter two-step test, experienced difficulty performing the 40 cm single-leg sit-to-stand test, and had higher 25-question Geriatric Locomotive Function Scale (GLFS-25) scores than those in the No-fall group. The results of the adjusted logistic regression analysis indicated that physical activity, higher GLFS-25 scores, and sedentary time of more than seven hours were all risk factors for falling or almost falling. Longer sedentary time is a new risk factor for falls among middle-aged women. It is necessary for people to be concerned with their sedentary behavior, such as by reducing or interrupting continuous sedentary time.

## 1. Introduction

According to the World Health Organization’s “World Health Statistics 2021,” people from Japan have a long average life expectancy worldwide at 81.5 years for men (second-longest globally) and 86.9 for women (longest globally). Additionally, healthy life expectancy, which is defined as having no limitations in daily life and no health problems, is 72.6 years for men and 75.5 years for women in Japan [1]. According to the Government of Japan’s Annual Report on the Ageing Society 2018, the difference in average life expectancy and healthy life expectancy is 8.84 years for men and 12.34 for women, with both men and women living inconvenient or bedridden lives for approximately 10 years. The main factors necessitating nursing care among older adults are frailty (13.8%) and fractures/falls (12.5%) [2]. According to the Health and Longevity Network, more than 90% of proximal femur, distal radius, and proximal humerus fractures are caused by falls, and preventing falls can reduce the number of older adults requiring nursing care [3].

The Japanese Orthopaedic Association advocates for the term “locomotive syndrome” to describe the decline in motor functions due to age-related muscle weakness, joint and spinal diseases, and osteoporosis, resulting in either having an increased risk of or becoming bedridden or requiring nursing care [4]. To close the gap between healthy life expectancy and average life expectancy, prevention of this locomotive syndrome is recommended [5]. Three tests are used to diagnose locomotive syndrome [6], namely, the 25-question Geriatric Locomotive Function Scale (GLFS-25) [7,8], which consists of 25 questions and requires participants to select the option (on a five-point scale) that best describes their physical and living conditions during the past month; the two-step test, which measures the distance of two steps; and the stand-up test, which requires one to stand up using only one leg from a seated height of 40, 30, 20, and 10 cm. There is a significant association between locomotive syndrome assessment and falls, with a reported 3.5-fold risk of falling when nonlocomotive syndrome is determined to be locomotive syndrome [9].

Falls are a recurring event. Older adults who had fallen had a fear of falling again [10,11], which led to reduced activity and was associated with reduced activities of daily living [12]. Actually, Rubenstein and Josephson reported that 50% of the older adults who had fallen in the past fell again [13]. For this reason, prevention of falls in old age is important. In order to prevent falls in old age, risk factors for falls in middle-aged and older adults need to be identified and prevented. However, there are few reports examining factors related to falls in middle-aged people. Since it has been reported that women are more likely than men to fall [14], the study aim was to determine the risk factors for falls among adult women between the ages of 40 and 65 years in Japan.

## 2. Methods

### 2.1. Study Sample

**Figure 1:**
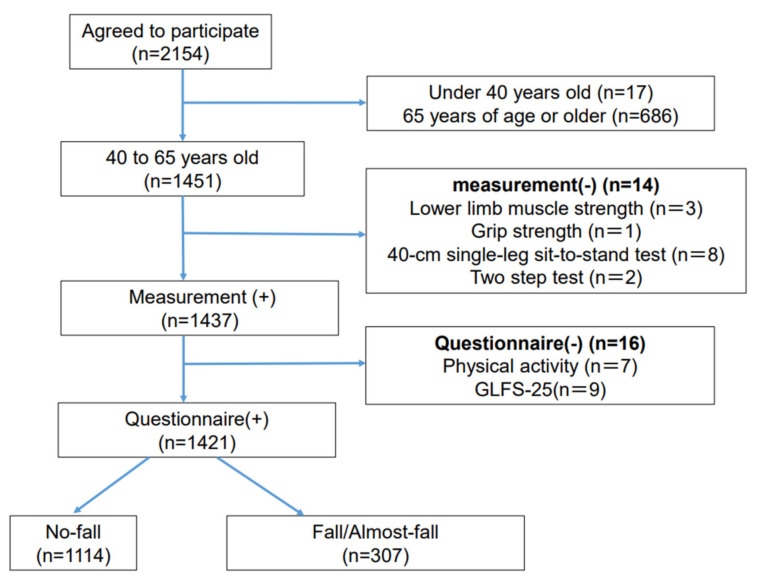
Flowchart detailing the enrollment process for the study.

Of a total of 2154 women who participated in the Japan Multi-Institutional Collaborative Cohort Study from Kyoto, Japan, between April 2016 and December 2017 [15,16,17], 17 women under 40 years old and 686 women aged 65 years and over were excluded from participating in this study; thus, 1451 women aged 40 to 64 years remained. Following the exclusion of those with incomplete measurements (lower-limb muscle strength, n = 3; grip strength, n = 1; 40 cm single-leg sit-to-stand test, n = 8; two-step test, n = 2) and questionnaires (GLFS-25, n = 9; physical activity, n = 7), 1421 participants were finally included in the study. The participants were divided into two groups (No-fall, Fall/Almost-fall) based on their answers to the question of whether they had fallen in the last month (Figure 1).

Before participating in this study, all participants provided written informed consent. Ethical approval was obtained from the Institutional Ethics Committee at Kyoto Prefectural University of Medicine (ERB-E-36), and this study was conducted in accordance with the principles of the World Medical Association Declaration of Helsinki.

### 2.2. Self-Administered Questionnaire

A self-administered questionnaire was used to collect data on the following: having fallen or almost fallen during the last month; having a history of diabetes mellitus (DM), hypertension (HT), or dyslipidemia (DL); using medications such as prescription stabilizers or sleeping pills; and the activities of daily living and GLFS-25 scores. The GLFS-25 consists of 25 items, including pain, activities of daily activity living, social functions, and mental health status during the last month. Participants who consumed alcohol or smoked cigarettes daily were categorized into the alcohol consumption and smoking groups, respectively.

Based on daily physical activity, the participants were classified into one of eight categories: none, <1 h/day, 1 to <3 h/day, 3 to <5 h/day, 5 to <7 h/day, 7 to <9 h/day, 9 to <11 h/day, and ≥11 h/day for heavy physical work, walking time, and standing time. Leisure time exercise was measured using the intensity, frequency per week, and duration of exercise. Physical activity was assessed in terms of metabolic equivalents and calculated based on heavy physical work, walking time, and leisure-time exercise [18]. Sedentary time not included as part of daily physical activity was divided into four categories: none to <5 h/day, 5 to <7 h/day, 7 to <9 h/day, and ≥9 h/day.

### 2.3. Measurements

Height and abdominal circumference were measured to the nearest 0.1 cm and weight to the nearest 0.1 kg. BMI was calculated as weight in kilograms divided by height in meters squared (kg/m^2^). Lower-limb muscle strength was measured using the Digital muscle strength system TP-776 (Toyo Physical Co. Ltd., Fukuoka, Japan); grip strength using the Smedley Hand Dynamometer (Matsumiya Medical Precision Machinery Co., Tokyo, Japan); and the two-step test using the maximum distance of two-steps-to-height ratio. The single-leg sit-to-stand test from a seat height of 40 cm consisted of using only one leg to stand up from a seated height of 40 cm with arms crossed in front of the chest to avoid recoil.

### 2.4. Medical Conditions

Participants being treated for HT, with a systolic blood pressure of ≥140 mmHg, or with diastolic blood pressure of ≥90 mmHg were considered to have hypertension. Blood pressure was measured with an HEM-759P Fuzzy Digital Automatic Blood Pressure Monitor (Omron Co., Kyoto, Japan) on the left arm in a sitting position after resting. Blood samples were collected from all participants. Triglyceride (TG), high-density lipoprotein cholesterol (HDL-C), low-density lipoprotein cholesterol (LDL-C), and glycated hemoglobin A1c (HbA1c) levels were all measured by enzymatic methods. Those being treated for DL and those with TG levels ≥ 150 mg/dL, HDL-C levels < 40 mg/dL, and LDL-C levels ≥ 140 mg/dL were considered to have DL. Participants who had developed and were being treated for DM, as well as those with HbA1c levels ≥ 6.5%, were considered to have DM.

### 2.5. Statistical Analyses

Continuous variables with a normal distribution were expressed as mean ± standard deviation. For categorical variables, the data were presented as values and percentages (%). All data were analyzed using SPSS Statistics 25.0 (IBM-SPSS Japan Inc., Tokyo, Japan) and a *p* < 0.05 was considered statistically significant. The differences in categorical variables between groups were analyzed using Pearson’s chi-square tests and the correlations between age and the various measurements were determined using Spearman’s correlation coefficient test.

A simple and multiple logistic regression analysis were used to determine the risk of falling. Three models were computed as follows: Model 1 was adjusted for age; Model 2 was adjusted for the covariates in Model 1 plus smoking, alcohol consumption, use of prescription stabilizers or sleeping pills, DM, HT, and DL; and Model 3 was adjusted for the covariates in Model 2 plus BMI, abdominal circumference, GLFS-25 score, lower-limb muscle strength, grip strength, physical activity, the 40 cm single-leg sit-to-stand test, and sedentary time. For sedentary time, the shortest group (none to <5 h/day) was considered to be the reference.

## 3. Results

The participants’ anthropometric, biochemical, and clinical characteristics are shown in Table 1. The No-fall group comprised 1114 participants aged 52.2 ± 7.0 years, and the Fall/Almost-fall group comprised 307 participants aged 52.5 ± 6.9 years. There were no differences in the incidence of DM, HT, or use of mood stabilizers and sleeping pills between the two groups. Compared to the No-fall group, BMI and abdominal circumference measurements and the prevalence of DL were significantly higher in the Fall/Almost-fall group (*p* < 0.05). Additionally, those in the Fall/Almost-fall group scored higher on the GLFS-25 than those in the No-fall group (*p* < 0.01), had a shorter two-step test, and experienced difficulty performing the single-leg sit-to-stand test (*p* < 0.01).

The correlations between age and the various measurements are shown in Table 2. Abdominal circumference, physical activity, and GLFS-25 score were positively associated with age (*p* < 0.01), although the two-step test and grip strength were negatively associated with age (*p* < 0.01). GLFS-25 score was positively associated with BMI and abdominal circumference (*p* < 0.01) and negatively associated with the two-step test (*p* < 0.01), lower limb muscle strength (*p* < 0.01), and grip strength (*p* < 0.05). Lower-limb muscle strength was positively associated with the two-step test and grip strength (*p* < 0.01).

The logistic regression analyses for the No-fall and Fall/Almost-fall groups are shown in Table 3. The unadjusted logistic regression analysis indicated that higher BMI (OR: 1.054, 95% CI: 1.016–1.093), abdominal circumference measurements (OR: 1.024, 95% CI: 1.010–1.037), and higher GLFS-25 scores (OR: 1.072, 95% CI: 1.049–1.095) were risk factors for falling in the Fall/Almost-fall group, and that the two-step test (OR: 0.211, 95% CI: 0.084–0.525), higher grip strength (OR: 0.968, 95% CI: 0.942–0.989), and the ability to perform the 40 cm single-leg sit-to-stand test (OR: 0.676, 95% CI: 0.519–0.882) were protective factors in the Fall/Almost-fall group. Similar results were obtained after adjusting for age, use of prescription stabilizers or sleeping pills, presence of DM, HT, DL, alcohol consumption, and smoking. However, when we added predictors such as BMI, abdominal circumference, GLFS-25 scores, lower-limb muscle strength, grip strength, the 40 cm single-leg sit-to-stand test, physical activity, and sedentary time to our analysis, we found that physical activity (OR: 1.018, 95% CI: 1.036–1.084), higher GLFS-25 scores (OR: 1.060, 95% CI: 1.036–1.084), and a sedentary time of 7–9 h (OR: 1.701, 95% CI: 1.117–2.592) and ≥9 h (OR: 1.680, 95% CI: 1.099–2.567) were all risk factors for falls.

## 4. Discussion

BMI, abdominal circumference measurements, and the presence of DL were significantly higher in the Fall/Almost-fall group than in the No-fall group. Ren et al. previously reported an association between obesity and falls in adults over 45 years of age [19], which is similar to the findings of this study, which showed a higher prevalence of obesity among the participants in the Fall/Almost-fall group. In particular, a greater abdominal circumference and the presence of dyslipidemia were frequently noted, suggesting that falls among the middle-aged are associated with visceral fat obesity. Additionally, many of the participants had high GLFS-25 scores, a short two-step test, and were unable to perform the 40 cm single-leg sit-to-stand test, suggesting that body pain and gait disturbances may make them more prone to falling. Similar to the results of this study, lower back and knee pain are reported to be associated with multiple falls [20,21]. It has been reported that older adults and women with pain have a slow walking speed [22,23]. In addition, pain not only slows walking speed but also makes walking unstable and leads to falls [24]. These finding suggest that reduction of physical pain and improvement of walking disorders are necessary to prevent falls in middle-aged and older adults.

Although there was no difference in lower-limb strength between the two groups, grip strength was significantly higher among participants in the No-fall group. Luisa et al. indicated that, while lower extremity muscle strength is associated with stumbling, high muscle strength could not prevent falls due to stumbling, and lower extremity muscle strength and balance can be associated or not [25]. Grip strength is associated with motor skills, lower limb strength, and dynamic balance [26,27]. In the older adults, decreased balance ability is a risk factor for falls, and grip strength is associated with physical function, including balance. In middle-aged people, the grip strength was lower in the Fall/Almost-fall group than in the No-fall group, suggesting that, as in the elderly, poor balance may be a risk for falls [28]. Since there is a negative correlation between age and grip strength, it is suggested that preventing the decline of grip strength and maintaining strength by not avoiding activities that require grip strength in daily life from a young age will be effective in preventing falls.

The presence of DM and medication use have also been reported to be associated with an increased risk of falls in older adults [29,30]. However, among the middle-aged women of this study, there was no difference in the presence of DM and medication use between the two groups. Furthermore, the results of Model 1 and Model 2 did not change when adjusted for age, diabetes, hypertension, dyslipidemia, stabilizers, and sleeping pills, indicating that illness and medication status do not affect falls in middle-aged women. However, when all factors, such as motor function, muscle strength, and physical activity, were adjusted for, factors related to obesity and muscle strength were no longer associated with falls. Contrarily, the amount of physical activity and a sedentary time of seven hours or more, which were not previously associated with GLFS-25 scores, became risk factors for falls.

In this study, pain remained one of the factors that increased the likelihood of falling, as GLFS-25 scores including the pain level were high. The fact that physical activity was a risk factor for falls may be explained by the positive correlation between age and physical activity. Older age and health consciousness influence the amount of physical activity one performs [31], and established exercise habits are more common in older-aged persons [32]. Therefore, after adjusting for all these factors, it is possible that physical activity levels pose a risk for falling. However, since no correlation was found between the amount of physical activity and grip strength, which is related to balance, it is believed that there is no relationship between a high physical activity level and good balance; therefore, performance of physical activity is not the only way to prevent falls. When all the factors were adjusted for, sedentary time became a risk factor for falls. Globally, Japan is reported to be one of the countries with the longest total sedentary time, at approximately seven hours per day [33]. In addition to falling, prolonged sedentary times increase mortality risk [34,35,36] and cognitive decline [37]. Sedentary time is a risk factor for obesity [38], whereby longer sedentary times are associated with higher visceral fat [39]. In this study, visceral obesity was considered a risk factor for falling, and with longer sedentary times, the risk of falling increases. It would be necessary to encourage people to stand instead of sitting on buses, trains, etc., when commuting to work, and to frequently interrupt their sitting time to walk or stand during breaks.

This study has several limitations. First, because it is a cross-sectional study, causality is not known. Second, the type of leisure-time exercise is not known. However, there are also no significant differences between the two groups with regard to the amount of physical activity or sedentary time. Furthermore, no difference was found in the amount of physical activity in leisure time (No-fall: 1.90 ± 2.6, Fall/Almost-fall: 1.99 ± 3.5, *p* = 0.598), and there is no association between leisure-time activity and falls. Therefore, it is unlikely that the type of leisure-time exercise has an effect on falls. Finally, we did not know the participants’ history of falls. In older adults, a history of falls is one of the strongest risk factors [40], and it has been reported that about half of older adults that have fallen have experienced multiple falls [13]. In this study, only six (1.9%) of the participants experienced multiple falls in one month. A history of falls may not be as strong a risk factor in middle-aged as it is in older adults, but we were unable to investigate this in the present study.

## 5. Conclusions

In this study, longer sedentary times were a new risk factor for falls among middle-aged women, particularly sedentary time of more than seven hours per day. To the best of our knowledge, this is the first time that sedentary time has been reported to be a risk factor for falling. For a healthy life expectancy, it is important to establish a lifestyle whereby sedentary time is reduced and standing or active time is increased. In the future, it is necessary to conduct longitudinal studies on the causal relationship between prolonged sedentary time and falls.

## Figures and Tables

**Table 1:** Anthropometric, biochemical, and clinical characteristics of the study participants, women aged 40 to 64.

	All	No Fall	Fall/Almost-Fall	*p*-Value
n (%)	1421	1114 (78.4)	307 (21.6)	
Age (years)	52.3 (±7.0)	52.2 (±7.0)	52.5 (±6.9)	0.560
BMI (kg/m^2^)	21.4 (±3.3)	21.3 (±3.2)	21.9 (±3.2)	**0.008**
AC (cm)	77.9 (±9.1)	77.5 (±8.8)	79.5 (±9.7)	**0.001**
SBP (mmHg)	122.0 (±18.1)	121.9 (±17.9)	122.3 (±18.8)	0.750
DBP (mmHg)	76.1 (±11.2)	75.9 (±11.0)	76.8 (±11.8)	0.214
HbA1c (%)	5.49 (±0.36)	5.48 (±0.34)	5.52 (±0.43)	0.063
HDL-C (mg/dL)	75.5 (±16.2)	76.2 (±16.2)	72.9 (±16.0)	**0.001**
LDL-C (mg/dL)	126.4 (±32.3)	125.5 (±32.0)	129.6 (±33.4)	**0.047**
TG (mg/dL)	83.6 (±52.8)	82.9 (±53.5)	86.4 (±49.9)	0.298
Physical activity (METs)	14.2 (±9.9)	14.1 (±9.6)	14.6 (±11.0)	0.487
GLFS-25 (point)	5.10 (±5.81)	4.53 (±5.22)	7.17 (±7.22)	**0.000**
Two-step test (m)	1.48 (±0.14)	1.48 (±0.14)	1.45 (±0.14)	**0.001**
Lower limb muscle strength (kg)	18.8 (±6.5)	18.9 (±6.5)	18.6 (±6.2)	0.410
Grip strength (kg)	31.2 (±5.3)	31.4 (±5.3)	30.5 (±5.5)	**0.004**
Current drinker (n, %)	763 (53.7)	594 (53.3)	169 (55.0)	0.591
Current smoker (n, %)	70 (4.9)	54 (4.8)	16 (5.2)	0.794
DM patient (n, %)	27 (1.9)	18 (1.6)	9 (2.9)	0.135
HT patient (n, %)	327 (23.0)	246 (22.1)	81 (26.4)	0.113
DL patient (n, %)	574 (40.4)	435 (39.0)	139 (45.3)	**0.049**
Prescription stabilizers or sleeping pills use (n, %)	71 (5.0)	50 (4.5)	21 (6.8)	0.094
40 cm single-leg sit-to-stand test successful (n, %)	989 (69.6)	796 (71.5)	193 (62.9)	**0.004**
Sedentary times				
None to <5 h/day (n, %)	369 (26.0)	300 (26.9)	69 (22.5)	0.148
5 to <7 h/day (n, %)	337 (23.7)	271 (24.3)	66 (21.5)	
7 to <9 h/day (n, %)	278 (19.6)	209 (18.8)	69 (22.5)	
≥9 h/day (n, %)	437 (30.7)	334 (30.0)	103 (33.6)	

Continuous variables are expressed as the mean (±SD). Prevalence was reported as a value (n) and percentage (%). Boldface indicates statistical significance (*p* < 0.05). DBP, diastolic blood pressure; DM, diabetes mellitus; GLFS-25, 25-question Geriatric Locomotive Function Scale; HbA1c, glycated hemoglobin A1c; HDL-C, high-density lipoprotein cholesterol; DL, dyslipidemia; HT, hypertension; LDL-C, low-density lipoprotein cholesterol; SBP, systolic blood pressure; TG, triglycerides; AC, abdominal circumference; METs, metabolic equivalents.

**Table 2:** Correlations between age and the various measurements using Spearman’s correlation coefficient.

	Age	BMI	Abdominal Circumference	Physical Activity	GLFS-25	Two-Step Test	Lower-Limb Muscle Strength	Grip Strength
Age	1	0.032	**0.119** **	**0.125** **	**0.111** **	**−0.074** **	0.027	**−0.223** **
BMI		1	**0.839** **	−0.013	**0.079** **	**−0.114** **	**0.263** **	**0.064** *
Abdominal circumference			1	−0.025	**0.116** **	**−0.159** **	**0.234** **	**0.058** *
Physical activity				1	−0.027	0.041	**0.066** *	−0.010
GLFS-25					1	**−0.151** **	**−0.074** **	**−0.135** **
Two-step test						1	**0.221** **	**0.226** **
Lower-limb muscle strength							1	**0.281** **
Grip strength								1

GLFS-25 = 25-Geriatric Locomotive Function Scale. Spearman’s correlation coefficient was used to measure these values. Boldface indicates statistical significance (* *p* < 0.05, ** *p* < 0.01).

**Table 3:** Crude and adjusted odds ratio for the risk of fall.

	Crude			Model 1 ^a^			Model 2 ^b^			Model 3 ^c^		
	OR	95% CI	*p*-Value	OR	95% CI	*p*-Value	OR	95% CI	*p*-Value	OR	95% CI	*p*-Value
BMI	**1.054**	**1.016–1.093**	**0.005**	**1.054**	**1.016–1.093**	**0.005**	**1.045**	**1.003–1.088**	**0.036**	0.985	0.905–1.072	0.729
Abdominal circumference	**1.024**	**1.010–1.037**	**0.001**	**1.023**	**1.010–1.037**	**0.001**	**1.021**	**1.006–1.036**	**0.007**	1.021	0.989–1.053	0.201
Two-step test	**0.211**	**0.084–0.525**	**0.001**	**0.213**	**0.085–0.533**	**0.001**	**0.236**	**0.093–0.597**	**0.002**	0.569	0.204–1.585	0.281
Lower-limb muscle strength	0.992	0.972–1.012	0.410	0.992	0.972–1.011	0.401	0.991	0.972–1.011	0.387	0.998	0.976–1.020	0.837
Grip strength	**0.968**	**0.942–0.989**	**0.004**	**0.965**	**0.941–0.989**	**0.005**	**0.966**	**0.942–0.991**	**0.007**	0.974	0.948–1.001	0.056
Physical activity	1.004	0.992–1.017	0.486	1.004	0.992–1.017	0.522	1.005	0.992–1.018	0.462	**1.018**	**1.036–1.084**	**0.020**
GLFS-25	**1.072**	**1.049–1.095**	**<0.001**	**1.072**	**1.049–1.095**	**<0.001**	**1.069**	**1.046–1.092**	**<0.001**	**1.060**	**1.036–1.084**	**<0.001**
40 cm single-leg sit-to-stand test	**0.676**	**0.519–0.882**	**0.004**	**0.677**	**0.517–0.888**	**0.005**	**0.700**	**0.533–0.921**	**0.011**	0.894	0.663–1.205	0.461
Sedentary times												
None to <5 h/day	Ref			Ref			Ref			Ref		
5 to <7 h/day	1.059	0.728–1.541	0.765	1.052	0.722–1.531	0.793	1.073	0.735–1.567	0.715	1.225	0.819–1.833	0.323
7 to <9h/day	1.435	0.984–2.094	0.061	1.430	0.980–2.086	0.064	1.411	0.964–2.063	0.076	**1.701**	**1.117–2.592**	**0.013**
9 h/day	1.341	0.952–1.888	0.093	1.313	0.953–1.891	0.092	1.332	0.943–1.882	0.103	**1.680**	**1.099–2.567**	**0.016**

^a^ Adjusted for age. ^b^ Adjusted for the covariates in Model 1 plus smoking, alcohol consumption, use of prescription stabilizers or sleeping pills, DM, HT, and DL. ^c^ Adjusted for the covariates in Model 2 plus BMI, abdominal circumference, the GLFS-25 score, lower limb muscle strength, grip strength, physical activity, the 40 cm single-leg sit-to-stand test, and sedentary time. GLFS-25, 25-Geriatric Locomotive Function Scale; DM, diabetes mellitus; HT, hypertension; DL, dyslipidemia.
